# Prevalence of depression, anxiety and stress and associated workplace stressors among healthcare workers in a tertiary hospital in Vietnam: A cross-sectional study

**DOI:** 10.1371/journal.pone.0347196

**Published:** 2026-04-20

**Authors:** Hoang-Phuong Nguyen, Thao Thi Phuong Nguyen, Ngoc Quang Nguyen, Thang Van Bui, Hai Anh Le, Van Thanh Hoang, Thanh Liem Nguyen

**Affiliations:** 1 Vinmec Research Institute of Stem Cell and Gene Technology, College of Health Sciences, VinUniversity, Vinhomes Ocean Park, Gia Lam, Hanoi, Vietnam; 2 Vinmec Times City International Hospital, Vinmec Healthcare System, Hanoi, Vietnam; 3 Vinmec Smart City International Hospital, Vinmec Healthcare System, Hanoi, Vietnam; Bach Mai Hospital, VIET NAM

## Abstract

**Background:**

Healthcare workers face disproportionate risks of depression, anxiety, and stress, yet evidence from private tertiary hospitals in Vietnam remains limited. We aimed to estimate the burden of psychological distress among healthcare workers and identify work-related correlates.

**Methods:**

We conducted a cross-sectional survey of healthcare workers at tertiary hospital in Hanoi (n = 505; physicians = 151, nurses = 304, others = 50). The validated Vietnamese DASS-21 assessed depression, anxiety, and stress severity. Sociodemographic, occupational, and psychosocial factors were collected. Group differences were tested using chi-squared and Kruskal-Wallis tests. Ordinal logistic regression analyses using complete cases (n = 478) examined correlates of each DASS domain, adjusting for demographic, occupational, and workplace interaction variables.

**Results:**

Overall, 30.3% of staff reported at least mild depressive symptoms, 28.7% at least mild anxiety symptoms, and 10.5% at least mild stress symptoms. Nurses exhibited the highest burden, with 34.9% reporting at least mild depression and 35.5% at least mild anxiety, compared with 21.2% and 16.0% among physicians and 30.0% and 26.0% among others, respectively. Stress symptoms were less prevalent (8.0% to 12.0%) and did not differ significantly across professional groups. In multivariable analyses, greater depression severity was associated with indefinite-term contracts and conflicts with patients or relatives; anxiety severity was associated with monthly income of 10–20 million VND and working in oncology; and stress severity was associated with caring for level II–III patients and contributing ≤50% of household income. Higher job satisfaction, supportive colleague relationships, perceived job stability, and greater continuing medical education exposure were associated with lower symptom severity across domains.

**Conclusions:**

In this private tertiary hospital, nurses reported a higher burden of depressive and anxiety symptoms than physicians, whereas stress symptoms were less common and varied little across professions. Conflict prevention, support in high-acuity settings, continuing education, and a supportive team climate may be priorities for workplace mental health support.

## Introduction

Healthcare workers are especially vulnerable to psychological disorders, which pose significant challenges for both individuals and health systems. Poor working conditions – such as excessive workloads, long shifts, and limited resources – contribute to increased mental health risks in this population [1]. These factors are linked to higher rates of absenteeism, staff turnover, medical errors, and reduced quality of patient care [2]. Global meta-analyses report that depression affects between 26.0% and 33.8% of healthcare workers, compared to just 4.0% to 5.7% in the general population, underscoring the disproportionate burden on this essential workforce [3–5]. The intense nature of healthcare work, involving high-pressure decisions, extended hours, and regular exposure to human suffering, further increases the risk of mental health conditions such as depression, anxiety, stress, and post-traumatic stress disorder [6–8].

Numerous studies indicate high rates of depression, anxiety, and stress among healthcare workers globally. For example, a systematic reviews and meta-analyses estimated that 22.8% experience depression and 23.2% experience anxiety [9]. These issues intensified during the COVID-19 pandemic, with anxiety symptoms reported in 35–48% and depressive symptoms in 28–38% of healthcare workers [10]. Burnout is also widespread, affecting 35% to 54.5% of this workforce, particularly among women, younger professionals, and nurses who appear more vulnerable [11,12]. Workplace stress, often measured using validated tools like the Depression Anxiety Stress Scales-21 (DASS-21), impacts between 22.8% and 47.9% of healthcare workers across diverse regions and practice settings [13,14].

In Vietnam, research on the mental health of healthcare workers remains limited. Existing studies, mostly conducted during the COVID-19 pandemic, reveal a significant psychological burden. For example, a cross-sectional survey in COVID-19 field hospitals (n = 542) found high rates of depression (46.8%), anxiety (38.3%), stress (60.2%), and post-traumatic stress disorder (21.2%) [15]. Similarly, elevated rates of depression (15.2%) and suicidal ideation (7.7%) among Vietnamese medical students point to early mental health risks in the healthcare training pipeline [16].

Despite these findings, few studies have examined the combined prevalence of depression, anxiety, and stress among healthcare workers in a large multi specialty general hospital, together with work-related factors. Previous research often focused on a single profession or one aspect of mental distress, limiting our understanding of overlapping symptoms and their occupational drivers. To address this gap, we conducted a cross-sectional study involving all permanent clinical staff at a tertiary hospital in Vietnam. The aim was to assess the prevalence of psychological distress and identify job-related stressors. By highlighting the scale of mental health challenges and their workplace links, our findings provide evidence to guide institutional interventions and policies that promote staff well-being and maintain high standards of patient care.

## Methods

### Study design, setting, and participants

We conducted a cross-sectional study of healthcare workers (physicians, nurses, and other professionals) at Vinmec Times City International Hospital, Hanoi, Viet Nam. Vinmec Times City is a high volume private multi-specialty general hospital providing comprehensive services across medical and surgical specialties, ranging from preventive and outpatient care to complex inpatient and oncology care. The hospital is part of a large private hospital network in Viet Nam and was selected because its broad specialty mix provides a diverse clinical workforce, and its standardized human resources and electronic medical record systems facilitate consistent staff sampling and data collection. Data were collected using a structured Vietnamese questionnaire covering sociodemographic, occupational, and psychosocial characteristics, alongside the 21-item Depression Anxiety Stress Scales (DASS-21).

### Data collection

All currently healthcare workers (physicians, nurses, and other hospital professionals) at Vinmec Times City International Hospital were invited to participate during the study period. Recruitment was coordinated through hospital internal channels (email and departmental notices) and directed invitees to a secure, web-based Vietnamese questionnaire. After reading an information sheet, participants provided electronic informed consent; only those indicating “Agree” were able to proceed and were retained for analysis. Of 515 staff recruited to participate, 4 declined and 6 did not complete the questionnaire; thus, 505 participants (151 physicians, 304 nurses, 50 others) completed the survey and were included in the descriptive analyses (completion rate 98.1%). Multivariable ordinal regression analyses were conducted using complete cases (n = 478).

### Measures

#### Outcomes – Depression, anxiety, and stress scale – 21 items.

Psychological distress was assessed using the Vietnamese DASS-21 which comprises three 7-item subscales, including Depression, Anxiety, and Stress, with responses scored 0–3; subscale sums are multiplied by two to yield 0–42. We classified severity using the conventional cut-points: Depression (Normal 0–9, Mild 10–13, Moderate 14–20, Severe 21–27, Extremely severe ≥28), Anxiety (Normal 0–7, Mild 8–9, Moderate 10–14, Severe 15–19, Extremely severe ≥20), and Stress (Normal 0–14, Mild 15–18, Moderate 19–25, Severe 26–33, Extremely severe ≥34). The DASS 21 was used as a screening measure of symptom severity and not as a diagnostic instrument; elevated scores indicate probable distress warranting clinical assessment [17]. The Vietnamese version of the DASS-21 has been culturally adapted and psychometrically validated in Vietnam since 2013; our study used this validated Vietnamese instrument [18].

#### Exposures and covariates.

*Sociodemographic:* age (years, derived from date of birth), gender (male/female), marital status (single/married/other), income (four levels, adjusted upward one level when monthly allowance ≥10–20 million VND), and household financial contribution (100%, > 75%, 50–75%, ≤ 50%).

*Occupational:* (1) professional group (physician/nurse/other); (2) years of professional experience; (3) tenure at current hospital – years; (4) department mapped to a harmonized specialty area and then to macro-groups ((i) Adult Medicine & Subspecialties, (ii) Acute/Critical & Perioperative Care; Pediatrics & Neonatology Women’s Health (OB-Gyn, IVF, Breast), (iii) Oncology, (iv) Orthopedics, Sports Medicine, & Rehabilitation, (v) Ancillary & Clinical Support (Imaging/Pharmacy/Nutrition/CSSD), (vi) Ambulatory & Preventive Services (OPD/Executive Health), (vii) Regenerative Medicine/Cell Therapy, (viii) Other Specialties, Administrative & Non-clinical, & Unknown/Other)); (5) employment contract (≤1 year, 1–3 years, > 3 years, indefinite); (6) highest patient acuity managed (Level I; Level II–III; none); working hours/week (<48, 48, > 48); (7) working days/week (<5.5, 5.5, > 5.5); (8) estimated overtime (main schedule and overtime, both binned categories).

*Perceptions and interactions:* perceived appropriateness of income and job for health, including perceived suitability of the current job for personal health, which was assessed with a single item, “How suitable is your current job for your health?”, with five response options ranging from “very suitable” to “not at all suitable”; self-rated work-related stress (Very high/High/Moderate/Low, later collapsed); colleague and line-manager relationships (4-level scales, with additional “No answer” level for missing); conflict frequencies with colleagues and with patients/families (ordinal with an explicit “None” level); job satisfaction (5-level and a binary “Satisfied [4–5] vs other”); perceived job stability (5-level and a binary “Stable [4–5] vs other”). When needed for transparency and to avoid data loss, ambiguous or missing free-text responses were retained as explicit categories (e.g., “No answer,” “Unclear/Not applicable”) and, where appropriate, entered into models as levels rather than deleted.

### Statistical analysis

Data were analysed using Stata/SE 19.5 (StataCorp), and figures were produced in R (RStudio). Distributional assumptions for continuous variables were assessed graphically and with the Shapiro–Wilk test. Categorical variables are presented as n (%), and continuous variables as mean ± SD. Comparisons across professional roles (physicians, nurses, and other healthcare workers) used the Kruskal–Wallis test for non-normally distributed continuous variables and Pearson’s χ² test for categorical variables, with Fisher’s exact test applied when expected cell counts were <5. Where overall group differences were statistically significant, post hoc pairwise comparisons were performed with appropriate adjustment for multiple testing. All tests were two sided with α = 0.05.

Associations with DASS-21 Depression, Anxiety, and Stress severity categories were examined using ordinal logistic regression with ordered outcome categories. Candidate predictors were pre-specified a priori based on a conceptual framework encompassing sociodemographic, occupational/workload, and psychosocial workplace factors. This framework, informed by occupational stress perspectives (job demands and job resources), guided the selection of predictors for inclusion in the fully adjusted models. To obtain a parsimonious model and reduce overfitting given the number of candidate predictors, we used a purposeful selection strategy: variables were screened using a liberal threshold (p ≤ 0.2) to avoid prematurely excluding potentially important covariates/confounders, and final models reported adjusted odds ratios (OR) and 95% confidence intervals (CI). As a sensitivity analysis, we additionally fitted theory-driven, fully adjusted models that included all a priori covariates (without stepwise selection) to evaluate the robustness of the findings. The proportional odds assumption was assessed using the Brant test for each final model, and no evidence of violation was observed in the final depression, anxiety, or stress models. To improve model stability in the depression and stress analyses, some sparse outcome and predictor categories were collapsed before fitting the final models. Multicollinearity was checked using variance inflation factors in the final models. No meaningful multicollinearity was observed. The maximum VIFs were 1.20 for the revised depression model, 2.24 for the anxiety model, and 1.52 for the stress model. Missing data were handled using complete case analysis unless otherwise stated. All tests were two-sided with α = 0.05, and statistical significance was defined as p < 0.05.

## Ethical approvals

The study protocol was reviewed and approved by the Ethics Committee in Biomedical Research at Vinmec International General Hospital and VinUniversity (VMEC-IRB) (Approval No. 102/2022/CN-HĐĐĐ VMEC; September 22, 2022; Hanoi, Vietnam). All procedures were conducted in accordance with the Declaration of Helsinki and applicable Vietnamese regulations. Participation was voluntary. Informed consent was obtained electronically: before accessing the questionnaire, participants were presented with an online information sheet and indicated their consent by selecting an “I agree to participate” option on the survey form. Survey responses were collected anonymously and were de-identified prior to analysis. Data were stored on secure, access-restricted servers.

## Results

A total of 505 healthcare workers completed the cross-sectional electronic survey (physicians = 151; nurses = 304; others = 50). Sociodemographic and socioeconomic characteristics are summarised in Table 1. Physicians were older than nurses and other healthcare workers (mean age 40.6 vs 33.1 and 32.5 years) and were more often male (47.7% vs 19.4–22.0%; both p < 0.001). Educational attainment was highest among physicians (residency/specialisation or postgraduate: 98.7%), whereas nurses and other staff mainly held college/university degrees (83.6% and 76.0%; p < 0.001). Nearly all physicians reported monthly incomes ≥40 million VND (98.7%) compared with 3.3% of nurses and 10.0% of other staff; by contrast, nurses and others clustered in the 10–40 million VND range (91.8% and 82.0%; p < 0.001). Physicians also more frequently contributed at least 75% of household income (45.0% vs 37.5% among nurses and 18.0% among other healthcare workers; p = 0.013) (Table 1).

**Table 1:** Sociodemographic and socioeconomic characteristics by professional group.

Characteristics	Physicians (n = 151)	Nurses (n = 304)	Others (n = 50)	P-value
**Age-years** (*mean ± SD)*	40.6 ± 10.2	33.1 ± 5.9	32.5 ± 6.5	0.000
**Gender** (%)				
Male	72 (47.7%)	59 (19.4%)	11 (22.0%)	0.000
Female	79 (52.3%)	245 (80.6%)	39 (78.0%)	
**Highest education level** (%)				
Intermediate level	0 (0.0%)	1 (0.3%)	1 (2.0%)	0.000
College/ University	2 (1.3%)	254 (83.6%)	38 (76.0%)	
Residency/ Specialization	38 (25.2%)	2 (0.7%)	1 (2.0%)	
Postgraduate	111 (73.5%)	47 (15.5%)	10 (20.0%)	
**Marital status** (%)				
Single	10 (6.6%)	48 (15.8%)	14 (28.0%)	0.003
Married	139 (92.1%)	252 (82.9%)	36 (72.0%)	
Other (divorced/ separated)	2 (1.3%)	4 (1.3%)	0 (0.0%)	
**Income** (%)				
< 10 million VND	0 (0.0%)	15 (4.9%)	4 (8.0%)	0.000
10–20 million VND	0 (0.0%)	151 (49.7%)	25 (50.0%)	
20–40 million VND	2 (1.3%)	128 (42.1%)	16 (32.0%)	
≥40 million VND	149 (98.7%)	10 (3.3%)	5 (10.0%)	
**Household financial contribution** (%)				
100%	18 (11.9%)	44 (14.5%)	3 (6.0%)	0.013
>75%	50 (33.1%)	70 (23.0%)	6 (12.0%)	
50-75%	57 (37.7%)	117 (38.5%)	25 (50.0%)	
≤50%	26 (17.2%)	73 (24.0%)	16 (32.0%)	

Occupational characteristics and workload varied substantially by professional group (Table 2). Physicians had the longest professional experience but the shortest tenure at the current hospital (15.9 ± 10.1 vs 11.1 ± 5.9 to 10.2 ± 5.9 years; 6.2 ± 3.9 vs 8.3 ± 3.6 to 7.4 ± 4.0 years). Nurses most commonly held indefinite-term contracts (89.8%), compared with physicians (68.9%) and others (82.0%), and were most represented in medicine-related clinical services, maternal and child health, and acute care and perioperative settings. They also managed the highest patient acuity (Level I or II–III: 77.6% vs 70.9% of physicians and 42.0% of others). Average working hours per week were broadly similar between physicians and nurses, whereas others more often worked ≤48 hours/week (76.0% vs 61.6% and 59.9%; p = 0.093). In contrast, physicians most often worked ≤5.5 days/week (72.2%), whereas nurses and others more often worked >5.5 days/week (65.5% and 70.0%, respectively; p < 0.001). Overtime patterns diverged: others most frequently reported no overtime (50.0%); physicians and nurses most often reported 0–48 h/wk of overtime (68.9% and 56.6%, respectively), while nurses also had the highest proportion reporting ≥48 h/wk (5.3%) and unclear/not applicable responses (9.5%) (p < 0.001). Regarding continuing medical education, physicians most commonly reported no/unclear exposure (78.1%). Compared with physicians, nurses and others had higher proportions reporting ≥36 h/year (24.7% and 34.0%, respectively), although no/unclear exposure remained the largest category in all three groups. Between-group differences were statistically significant for years of professional experience and tenure at the current hospital, in addition to specialty area, contract type, patient acuity, average working days, overtime, and CME training; only average working hours per week did not differ significantly (p = 0.093).

**Table 2:** Occupational characteristics and workload by professional group.

Characteristics	Physicians (n = 151)	Nurses (n = 304)	Others (n = 50)	P-value
**Years of professional experience** *(mean ± SD)*	15.9 ± 10.1	11.1 ± 5.9	10.2 ± 5.9	0.000
**Tenure at current hospital – years** (mean ± SD)	6.2 ± 3.9	8.3 ± 3.6	7.4 ± 4.0	0.000
**Clinical/Professional specialty area** (%)				
Medicine-related clinical services	48 (31.8%)	90 (29.6%)	8 (16.0%)	0.000
Acute care and perioperative	15 (9.9%)	54 (17.8%)	0 (0.0%)	
Maternal and child health	39 (25.8%)	79 (26%)	12 (24.0%)	
Specialty procedural and advanced therapies	16 (10.6%)	48 (15.8%)	8 (16.0%)	
Other	33 (21.9%)	33 (10.9%)	22 (44.0%)	
**Type of employment contract** (%)				
Fixed-term	1 (0.7%)	6 (2.0%)	0 (0.0%)	0.000
Indefinite-term	104 (68.9%)	273 (89.8%)	41 (82.0%)	
**Highest patient acuity level managed** (%)				
Level I (critical; continuous monitoring; fully dependent)	53 (35.1%)	138 (45.4%)	12 (24.0%)	0.000
Level II–III (severely ill)	54 (35.8%)	98 (32.2%)	9 (18.0%)	
None (no care of critically ill patients)	44 (29.1%)	68 (22.4%)	29 (58.0%)	
**Average working hours per week** (%)				
≤48 hours/week	93 (61.6%)	182 (59.9%)	38 (76.0%)	0.093
>48 hours/week	58 (38.4%)	122 (40.1%)	12 (24.0%)	
**Average working days per week** (%)				
≤5.5 days/week	109 (72.2%)	105 (34.5%)	15 (30%)	0.000
>5.5 days/week	42 (27.8%)	199 (65.5%)	35 (70%)	
**Overtime hours per week** (%)				
0 h/wk	41 (27.2%)	87 (28.6%)	25 (50%)	0.000
0–48 h/wk	104 (68.9%)	172 (56.6%)	23 (46%)	
≥48 h/wk	2 (1.3%)	16 (5.3%)	2 (4%)	
Unclear/Not applicable	4 (2.6%)	29 (9.5%)	0 (0%)	
**Other/CME training hours in the past 12 months** (%)				
<36 h/year	13 (8.6%)	11 (3.6%)	2 (4.0%)	0.002
≥36 h/year	16 (10.6%)	75 (24.7%)	17 (34.0%)	
No/Unclear	118 (78.1%)	210 (69.1%)	27 (54.0%)	

Perceptions and outcomes varied by professional group. A majority judged their income as inappropriate relative to their abilities, with physicians least likely to rate their income as appropriate (4.6%), compared with nurses (39.1%) and others (18.0%; p < 0.001). In contrast, most participants considered their current job suitable for their health, although this proportion was lowest among nurses (86.2%) compared with physicians (96.0%) and others (98.0%; p = 0.001). High/very high work stress was common in all groups (physicians 68.9%, nurses 76.3%, others 64.0%) with no significant differences (p = 0.081). Collegial relationships were largely positive (good/very good: 92.1% physicians, 86.2% nurses, 82.0% others; p = 0.094), and conflicts with line managers were infrequent (p = 0.551). In contrast, physicians reported more conflicts with patients/families than nurses and others (29.8% vs 18.8% and 10.0%; p = 0.003). Job satisfaction and perceived job stability were highest among physicians (68.2% satisfied; 60.3% stable) and lowest among nurses (37.2% satisfied; 40.1% stable; both p < 0.001) (Table 3).

**Table 3:** Perceptions, workplace interactions, and work-related outcomes.

Characteristics	Physicians (n = 151)	Nurses (n = 304)	Others (n = 50)	P-value
**Perceived appropriateness of income relative to abilities** (%)				
Suitable	7 (4.6%)	119 (39.1%)	9 (18.0%)	0.000
Not suitable	144 (95.4%)	185 (60.9%)	41 (82.0%)	
**Perceived appropriateness of current job for health** (%)				
Suitable	145 (96.0%)	262 (86.2%)	49 (98.0%)	0.001
Not suitable	6 (4.0%)	42 (13.8%)	1 (2.0%)	
**Self-rated level of work-related stress** (%)				
Very high/High	104 (68.9%)	232 (76.3%)	32 (64%)	0.081
Moderate/Low	47 (31.1%)	72 (23.7%)	18 (36%)	
**Colleague relationship** (%)				
Average/Not good	12 (7.9%)	42 (13.8%)	9 (18.0%)	0.094
Good/Very good	139 (92.1%)	262 (86.2%)	41 (82.0%)	
**Frequency of conflicts with colleagues** (%)				
None (no conflicts)	91 (60.3%)	181 (59.5%)	29 (58%)	0.960
Any conflicts	60 (39.7%)	123 (40.5%)	21 (42%)	
**Relationship with line manager** (%)				
Good/Very good	49 (32.5%)	76 (25.0%)	14 (28.0%)	0.118
Average/Not good	11 (7.3%)	47 (15.5%)	7 (14.0%)	
No answer	91 (60.3%)	181 (59.5%)	29 (58.0%)	
**Frequency of conflicts with line manager** (%)				
None (no conflicts)	121 (80.1%)	255 (83.9%)	40 (80%)	0.551
Any conflicts	30 (19.9%)	49 (16.1%)	10 (20%)	
**Relationship with patient/family** (%)				
Good/Very good	29 (19.2%)	35 (11.5%)	8 (16.0%)	0.055
Average/Not good	1 (0.7%)	14 (4.6%)	2 (4.0%)	
No answer	121 (80.1%)	255 (83.9%)	40 (80.0%)	
**Frequency of conflicts with patients/family** (%)				
None (no conflicts)	106 (70.2%)	247 (81.2%)	45 (90%)	0.003
Any conflicts	45 (29.8%)	57 (18.8%)	5 (10%)	
**Satisfaction with current job** (%)				
Neutral/Not satisfied	48 (31.8%)	191 (62.8%)	22 (44.0%)	0.000
Satisfied	103 (68.2%)	113 (37.2%)	28 (56.0%)	
**Perceived job stability-few position changes/rotations** (%)				
Not stable	60 (39.7%)	182 (59.9%)	22 (44.0%)	0.000
Stable	91 (60.3%)	122 (40.1%)	28 (56.0%)	

**Fig 1:**
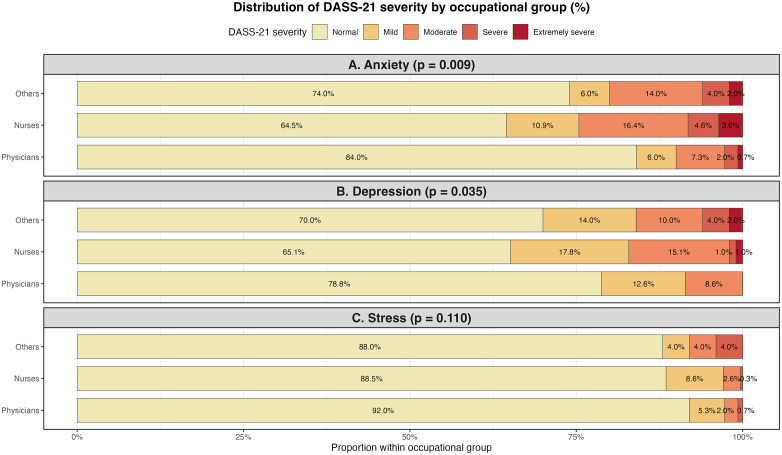
Distribution of DASS-21 severity categories by professional group.

The overall and group-specific prevalence of at least mild depression, anxiety and stress is shown in Table 4 and Fig 1. Table 4 presents the prevalence of depression, anxiety, and stress according to severity levels across occupational groups. While the majority of respondents were classified as “normal” across all domains, clinically meaningful symptoms remained prevalent. Nurses demonstrated the highest burden of depressive and anxiety symptoms (34.9% and 35.5%, respectively), compared with physicians (21.2% and 16.0%) and other healthcare workers (30.0% and 26.0%). Stress-related symptoms were less frequent overall and showed little variation across groups, ranging from 8.0% in physicians to 12.0% in other healthcare workers.

**Table 4:** Prevalence of depression, anxiety, and stress among physicians, nurses, and other healthcare workers (% within each group).

Psychological domain	Severity category	Physicians (%)	Nurses (%)	Others (%)
**Depression**	Normal	78.8	65.1	70.0
	With disorder (mild or more severe)	21.2	34.9	30.0
**Anxiety**	Normal	84.0	64.5	74.0
	With disorder (mild or more severe)	16.0	35.5	26.0
**Stress**	Normal	92.0	88.5	88.0
	With disorder (mild or more severe)	8.0	11.5	12.0

***Note****:* Percentages are based on DASS 21 severity categories within each occupational group. “At least mild symptoms” includes mild, moderate, severe, and extremely severe levels and reflects screening defined symptom severity rather than clinical diagnosis.

The most substantial between-group difference was observed for anxiety: nurses exceeded physicians by 19.5 percentage points (35.5% vs. 16.0%), representing approximately a 2.2-fold higher prevalence. For depression, the gap was somewhat smaller but still clinically meaningful, with nurses exceeding physicians by 13.7 percentage points (~1.65-fold higher). Stress-related symptoms showed the smallest occupational variation, though the pattern remained consistent: other healthcare workers (12.0%) and nurses (11.5%) both exceeded physicians (8.0%).

Fig 1 shows that depression and anxiety severity differed by profession (p = 0.035 and p = 0.009). Physicians most often screened “normal” for both depression (78.8%) and anxiety (84.0%), whereas nurses had lower “normal” rates (65.1% and 64.5%, respectively) and higher mild-moderate (and some severe) levels. The other healthcare worker group generally showed an intermediate pattern for depression and anxiety, with small severe/extremely severe proportions. Stress severity did not differ significantly across groups (p = 0.110); ≥ 88% in each group were “normal,” and severe/extreme stress was rare.

In the final multivariable ordinal logistic regression models shown in Table 5, depression severity was higher among staff on indefinite-term contracts (aOR 2.40, 95% CI 1.22–4.71) and among those reporting conflicts with patients/families, with the strongest associations for “almost never” (aOR 6.66, 2.38–18.64) and “occasionally” (aOR 4.17, 1.87–9.30), and a weaker trend for “rarely” (aOR 1.79, 0.91–3.55). Lower depression severity was associated with job satisfaction (aOR 0.45, 0.25–0.79), good or very good colleague relationships (aOR 0.47, 0.26–0.85), perceived job stability (aOR 0.48, 0.29–0.80), and a dose response pattern with lower self rated work stress. Depression severity was also lower among staff who perceived their current job as not suitable for their health (aOR 0.55, 0.33–0.92). For anxiety, greater severity was linked to monthly income of 10–20 million VND (aOR 1.73, 1.10–2.73) and working in oncology (aOR 2.62, 1.02–6.75), whereas no clear association was observed for relationship or conflicts with line managers in the final anxiety model. Anxiety severity was lower among staff who were satisfied with their job (aOR 0.59, 0.35–0.98), reported more CME exposure (24–36 h/year: aOR 0.16, 0.04–0.69; ≥ 36 h/year: aOR 0.33, 0.12–0.92; no/unclear vs 0–12 h/year: aOR 0.25, 0.10–0.66), perceived their job as stable (aOR 0.52, 0.32–0.85), and rated their work stress lower. Stress severity was higher among those managing Level II–III patients (aOR 2.22, 1.08–4.57) and contributing ≤50% of household income (aOR 2.18, 1.02–4.63), with a non-significant trend in ambulatory/preventive services (aOR 3.45, 0.93–12.73). Lower stress was observed among staff who perceived their current job as not suitable for health (aOR 0.37, 0.18–0.79), those reporting lower self-rated work stress, and those with ≥36 h/year of CME (aOR 0.16, 0.04–0.62) or no/unclear CME exposure (aOR 0.31, 0.10–0.96) (Table 5). Fully adjusted sensitivity analyses are presented separately in S1 Table.

**Table 5:** Ordinal regression analysis of related factors of depression, anxiety, and stress.

Predictor (reference)	aOR (95% CI)
**A. Depression model**	
**Gender (ref: male)**	
Female	1.59* (0.93–2.72)
**Marital status (ref: single)**	
Married	0.54* (0.29–1.01)
**Household financial contribution (ref: 100%)**	
50–75%	1.46* (0.94–2.26)
**Type of employment contract (ref: less than 1 year)**	
Indefinite-term (open-ended/permanent)	2.40** (1.22–4.71)
**Frequency of conflicts with patients/family (ref: none)**	
Almost never	6.66*** (2.38–18.64)
Rarely	1.79* (0.91–3.55)
Occasionally	4.17*** (1.87–9.30)
**Satisfaction with current job (ref: neutral/not satisfied)**	
Satisfied	0.45*** (0.25–0.79)
**Average working days per week (ref: < 5.5 days/week)**	
>5.5 days/week	0.70 (0.45–1.08)
**Overtime hours per week (ref: 0 h/wk)**	
≥48 h/wk	1.87 (0.72–4.86)
**Perceived suitability of current job for health (ref: suitable)**	
Not suitable	0.55** (0.33–0.92)
**Self-rated level of work-related stress (ref: very high)**	
High	0.46** (0.24–0.87)
Moderate/Low	0.27*** (0.13–0.57)
**Other/CME training hours in the past 12 months (ref: 0–12 h/year)**	
12–24 h/year	2.60 (0.66–10.31)
24–36 h/year	0.40 (0.11–1.39)
**Colleague relationship (ref: average/not good)**	
Good/Very good	0.47** (0.26–0.85)
**Perceived job stability (ref: not stable)**	
Stable	0.48*** (0.29–0.80)
**B. Anxiety model**	
**Marital status (ref: single)**	
Married	0.62 (0.34–1.11)
**Income (ref: < 10 million VND)**	
10–20 million VND	1.73** (1.10–2.73)
**Clinical/Professional specialty area (ref: Adult Medicine & Subspecialties)**	
Oncology	2.62** (1.02–6.75)
**Type of employment contract (ref: less than 1 year)**	
1–3 years	0.63 (0.31–1.27)
**Satisfaction with current job (ref: neutral/not satisfied)**	
Satisfied	0.59** (0.35–0.98)
**Overtime hours per week (ref: 0 h/wk)**	
Unclear/Not applicable	1.82 (0.71–4.68)
**Self-rated level of work-related stress (ref: very high)**	
High	0.36*** (0.20–0.67)
Moderate/Low	0.22*** (0.11–0.44)
**Other/CME training hours in the past 12 months (ref: 0–12 h/year)**	
24–36 h/year	0.16** (0.04–0.69)
≥36 h/year	0.33** (0.12–0.92)
No/Unclear	0.25*** (0.10–0.66)
**Relationship with line manager (ref: good/very good)**	
Average/Not good	1.46 (0.81–2.63)
**Frequency of conflicts with line manager (ref: none)**	
Rarely	1.75 (0.88–3.48)
**Perceived job stability (ref: not stable)**	
Stable	0.52*** (0.32–0.85)
**C. Stress model**	
**Gender (ref: male)**	
Female	0.55 (0.26–1.19)
**Household financial contribution (ref: 100%)**	
≤50%	2.18** (1.02–4.63)
**Clinical/Professional specialty area (ref: Adult Medicine & Subspecialties)**	
Ambulatory & Preventive Services (OPD/Executive Health)	3.45* (0.93–12.73)
Other Specialties, Administrative & Non-clinical, & Unknown/Other	2.18 (0.79–5.96)
**Type of employment contract (ref: less than 1 year)**	
1–3 years	0.42 (0.11–1.55)
**Highest patient acuity level managed (ref: level I)**	
Level II–III	2.22** (1.08–4.57)
**Overtime hours per week (ref: 0 h/wk)**	
Unclear/Not applicable	2.48 (0.70–8.75)
**Perceived appropriateness of current job for health (ref: suitable)**	
Not suitable	0.37** (0.18–0.79)
**Self-rated level of work-related stress (ref: very high)**	
High	0.33*** (0.14–0.75)
Moderate/Low	0.14*** (0.04–0.46)
**Other/CME training hours in the past 12 months (ref: 0–12 h/year)**	
12–24 h/year	2.61 (0.46–14.88)
≥36 h/year	0.16*** (0.04–0.62)
No/Unclear	0.31** (0.10–0.96)
**Frequency of conflicts with colleagues (ref: none)**	
Rarely	1.60 (0.74–3.42)
**Relationship with line manager (ref: good/very good)**	
Average/Not good	0.43** (0.18–1.00)
**Frequency of conflicts with line manager (ref: none)**	
Rarely	2.21 (0.85–5.80)
Occasionally	3.09* (0.99–9.65)
**Satisfaction with current job (ref: neutral/not satisfied)**	
Satisfied	0.52 (0.23–1.21)

**Note.** Adjusted odds ratios (aORs) and 95% confidence intervals (CIs) are from multivariable ordinal logistic regression models (proportional odds). ***Abbreviations:*** aOR, adjusted odds ratio; CI, confidence interval; CME, continuing medical education; OPD, outpatient department; OR, odds ratio; Ref, reference category; VND, Vietnamese dong.

*** p < 0.01, ** p < 0.05, * p < 0.1

## Discussion

This study provides the first evidence from Vietnam examining depression, anxiety, and stress among physicians, nurses, and other healthcare workers in a large private tertiary hospital during the post–COVID-19 period. We observed a clear gradient in mental-health burden across professional groups: 34.9% of nurses screened positive for depression and 35.5% for anxiety, compared with 21.2% and 16.0% among physicians and 30.0% and 26.0% among other staff. It is important to note that the DASS 21 is a validated self report scale measuring the severity of depression, anxiety, and stress symptoms and is widely used as a screening instrument rather than a diagnostic tool. Thus, elevated DASS 21 scores should be interpreted as indicating probable psychological distress warranting further clinical evaluation, rather than definitive clinical diagnoses. In this context, the DASS 21 may be useful for occupational mental health screening, whereas diagnostic confirmation and treatment decisions should be based on formal clinical assessment. This pattern accords with international evidence showing that nurses – particularly those in high-acuity settings – experience more frequent and severe psychological morbidity than physicians and other health professionals [19–21]. Recent meta-analyses similarly indicate that nurses have the highest relative risk of depression among healthcare workers (RR = 3.5, 95% CI 1.3–9.6), and large-scale studies report that 47% of nurses versus 32% of physicians meet criteria for high burnout; ICU nurses have more than fivefold higher odds of depressive symptoms than ICU physicians (OR = 5.33) [22]. Consistent findings have been reported in China, where the prevalence of anxiety and depressive disorders was 31.0% and 53.3% in physicians and 30.8% and 47.9% in nurses [21].

Several occupational features may help explain these disparities. Nurses provide continuous direct care, have more frequent patient contact, and often work rotating schedules, all of which may intensify psychological strain; physicians, despite heavy responsibility, may have greater autonomy and more structured support [23]. Staff in ‘other’ roles showed intermediate outcomes, possibly reflecting less direct clinical exposure and lower reported conflict with patients/families [24]. Importantly, stress levels differed little between physicians and nurses, suggesting that common frontline stressors, including high workloads, long shifts, and patient volume, may be pervasive across roles [25,26]. This pattern is consistent with the DASS-Stress subscale capturing general stress reactivity that may be broadly elevated in high-intensity hospital settings [25]. Prior studies likewise report smaller occupational differences for stress than for depression or anxiety, with nurses continuing to show a disproportionate burden of the latter outcomes [24,27,28].

In multivariable analyses, several workplace factors were associated with mental health severity. Staff on indefinite-term contracts had higher odds of more severe depression than those in the reference contract category (<1 year) (aOR = 2.40; 95% CI: 1.22–4.71). Because this finding contrasts with much of the occupational health literature, it should be interpreted cautiously and not as evidence of a causal effect of contract type itself [29,30]. Residual confounding is possible, as permanent staff may differ in age, tenure, supervisory roles, and cumulative workload burden [19,31]. Rather than suggesting that job security is harmful, this result may indicate that some long-tenured staff face greater role-related strain and may benefit from closer monitoring and tailored support [32].

Conflicts with patients and family members were among the strongest correlates of higher depression severity in the final model, consistent with evidence linking workplace conflict and violence to poorer mental health among healthcare workers [33,34]. Repeated difficult interactions may erode psychological safety and increase emotional exhaustion, a pattern also reflected in Vietnamese data [2]. These findings support stronger conflict-prevention measures, communication training, and rapid-response support for frontline staff. By contrast, anxiety severity was more strongly associated with mid-range income and working in oncology, while no clear association was observed for relationship or conflicts with line managers.

Assignments involving level II–III patients were associated with higher stress. Caring for severely ill patients often require sustained vigilance, rapid decisions, and repeated exposure to suffering, all of which may heighten psychological strain. This interpretation is consistent with prior studies showing greater stress among staff working in high-acuity settings [35,36]. Adequate staffing, rotation policies, and protected recovery time may therefore be important in these units.

Protective associations were also identified, although these were somewhat domain-specific: job satisfaction was associated with lower depression and anxiety severity; good or very good colleague relationships were associated with lower depression severity; perceived job stability was associated with lower depression and anxiety severity; and greater CME exposure was associated with lower anxiety severity, with selected CME categories also associated with lower stress severity [20,35,37,38]. These findings suggest that workplace mental health interventions should address both interpersonal and structural stressors, including patient-facing conflict, support in high-acuity settings, and opportunities for professional development.

Some findings, however, require cautious interpretation. The association between indefinite term contracts and higher depression severity contrasts with much of the occupational health literature, in which employment stability is often considered protective. This pattern may reflect residual confounding by factors not fully captured in the model, such as age, seniority [39], supervisory or leadership responsibilities [40], administrative burden [41], or cumulative workload among longer tenured staff [42]. Similarly, the inverse association between perceiving one’s current job as “not suitable for health” and lower depression and stress severity was unexpected and should also be interpreted cautiously. Because this construct was assessed using a single self reported item, the observed association may partly reflect differential interpretation of the item, response bias, or measurement artefact [43]. It may also reflect adaptation or coping processes, whereby staff who explicitly recognize the health burden of their work differ in how they appraise or manage workplace stressors [44]. Residual confounding by unmeasured occupational factors, such as workload composition, role modification, seniority, or supervisory responsibilities, also cannot be excluded. In addition, the inverse association between lower self-rated work stress and lower DASS-21 symptom severity should be interpreted carefully because self-rated work stress may partly overlap conceptually with depression, anxiety, and stress symptom reporting. Given the cross-sectional design, these findings should not be interpreted as evidence that indefinite-term contracts increase depression severity or that poorer perceived job-health fit protects against depression or stress. Overall, the results support a hospital mental health strategy that extends beyond individual screening to include safer patient-facing work environments, stronger team support, appropriate staffing and recovery in high-acuity settings, and protected opportunities for continuing education.

This study has several strengths. First, it enrolled a relatively large sample of 505 healthcare workers from a leading private tertiary multi-specialty hospital, providing sufficient statistical power and a comprehensive snapshot of mental health in an urban private setting. The use of a standardized electronic survey, supported by robust hospital IT infrastructure, likely reduced data entry errors and other systematic biases. Second, we employed the Vietnamese DASS-21, which has been culturally and psychometrically validated, enabling more reliable assessment of depression, anxiety and stress and facilitating comparison with international literature. Third, we applied a structured analytic strategy, combining detailed descriptive analyses with hierarchical multivariable regression to account for demographic, occupational and psychosocial factors when estimating independent associations.

Several limitations should also be acknowledged. The cross-sectional design precludes causal inference, and reliance on self-report may introduce social desirability and recall bias. Although the survey completion rate among recruited staff was high (98.1%), participation was voluntary, and the electronic survey design may still be subject to selection and non-response bias. Staff with higher workloads or greater psychological distress may have been less likely to participate in or complete the questionnaire, potentially biasing prevalence estimates in either direction. As this was a single-center study conducted in a large urban private tertiary hospital, the findings may not be directly generalizable to public hospitals or rural settings, where staffing structures, patient volume, case mix, resource constraints, and workplace organization may differ substantially. In addition, multivariable analyses were based on complete cases (n = 478), which may introduce bias if covariate missingness was not completely at random. A stepwise selection strategy was used primarily for parsimony; however, data-driven variable selection can produce unstable models, increase the risk of overfitting, and exclude relevant confounders, resulting in residual confounding. Although we additionally fitted theory-driven fully adjusted models as a sensitivity analysis, the identified associations should still be interpreted cautiously and confirmed in independent, preferably multicentre, studies. Future research should employ multicenter longitudinal cohorts across public and private hospitals in both urban and rural areas to establish temporal associations and evaluate workplace interventions, such as workload adjustment and communication training. Mediation and moderation analyses, for example, using structural equation modelling, could clarify whether factors such as job satisfaction or peer relationships mediate the impact of workplace stressors on DASS-21 outcomes and whether professional role modifies these associations. In-depth qualitative work is also needed to explore experiences of patient-family conflict, contract pressures, and shift work, and to inform the co-design of human-centred interventions, including timely psychological support following critical incidents.

## Conclusion

Nurses reported a greater burden of depressive and anxiety symptoms than physicians, whereas stress was less prevalent and showed limited variation across professional groups. Several workplace and occupational factors were associated with mental health severity, although some findings, particularly the association with indefinite-term contracts, should be interpreted cautiously. Job satisfaction, supportive colleague relationships, perceived job stability, and greater continuing medical education exposure were associated with lower symptom severity. These findings suggest potential areas for intervention, including conflict prevention, staff support in high-acuity settings, and continued investment in education and team climate. Findings are limited by the cross-sectional, single-centre, self-reported design. Multicentre longitudinal and qualitative studies are needed to confirm these patterns and guide targeted workplace interventions.

## Supporting information

S1 Table Fully adjusted ordinal regression results. (DOCX)
