# Supplementary material for: Prevalence of depression, anxiety and stress and associated workplace stressors among healthcare workers in a tertiary hospital in Vietnam: A cross-sectional study
Source: PLoS One. 2026 Apr 20;21(4):e0347196. doi: 10.1371/journal.pone.0347196 (PMC13094995; doi:10.1371/journal.pone.0347196)
Supplement: S1 Table — (DOCX) [file pone.0347196.s001.docx]

**Supplementary Table 1. Fully adjusted ordinal regression results**

|  | **Depression** | **Anxiety** | **Stress** |
| --- | --- | --- | --- |
|  | **OR (95% CI)** | **OR (95% CI)** | **OR (95% CI)** |
| **Age (years)** |  |  |  |
| Per 1-unit increase | 0.99 (0.89-1.09) | 1.02 (0.92-1.12) | 0.95 (0.85-1.07) |
| **Gender** |  |  |  |
| Male | Ref | Ref | Ref |
| Female | 0.84 (0.43-1.63) | 1.73 (0.87-3.44) | 0.43 (0.13-1.44) |
| **Education level** |  |  |  |
| High school or below | Ref | Ref | Ref |
| College / University | 0.07* (3.25e-3-1.52) | 1.55e+6*** (9.28e+4-2.58e+7) | 8.04e+5*** (627.1-1.03e+9) |
| Residency / Specialization | 0.09 (2.63e-3-2.91) | 1.07e+6*** (7.32e+4-1.57e+7) | 1.91e+5*** (24.51-1.48e+9) |
| Postgraduate | 0.15 (5.24e-3-4.17) | 1.08e+6*** (8.48e+4-1.38e+7) | 1.59e+6*** (309.62-8.20e+9) |
| **Marital status** |  |  |  |
| Single | Ref | Ref | Ref |
| Married | 0.43** (0.20-0.91) | 0.65 (0.29-1.46) | 0.58 (0.19-1.72) |
| Other (divorced / separated) | 0.88 (0.11-6.97) | 4.46e-7*** (7.20e-8-2.76e-6) | 1.16e-6*** (7.19e-8-1.86e-5) |
| **Monthly income** |  |  |  |
| <10 million VND | Ref | Ref | Ref |
| 10–<20 million VND | 4.19 (0.50-34.83) | 1.31 (0.22-7.95) | 2.23 (0.13-39.42) |
| 20–<40 million VND | 3.15 (0.32-31.22) | 0.98 (0.13-7.41) | 3.57 (0.13-95.25) |
| ≥40 million VND | 1.65 (0.12-22.79) | 0.97 (0.09-10.2) | 2.72 (0.05-142.54) |
| **Household financial contribution** |  |  |  |
| 100% | Ref | Ref | Ref |
| >75% | 1.20 (0.51-2.85) | 1.20 (0.51-2.82) | 1.38 (0.36-5.35) |
| 50–75% | 1.36 (0.64-2.90) | 1.54 (0.68-3.51) | 0.90 (0.25-3.22) |
| ≤50% | 0.85 (0.38-1.92) | 1.31 (0.56-3.09) | 1.93 (0.49-7.65) |
| **Tenure in profession (years)** |  |  |  |
| Per 1-unit increase | 0.98 (0.89-1.09) | 0.97 (0.88-1.07) | 1.00 (0.90-1.12) |
| **Tenure at Vinmec (years)** |  |  |  |
| Per 1-unit increase | 1.05 (0.95-1.16) | 1.00 (0.90-1.11) | 0.98 (0.84-1.15) |
| **Specialty area (macro group)** |  |  |  |
| Adult Medicine & Subspecialties | Ref | Ref | Ref |
| Acute/Critical & Perioperative Care | 1.74 (0.75-4.06) | 1.67 (0.68-4.13) | 0.64 (0.16-2.55) |
| Pediatrics & Neonatology | 1.12 (0.43-2.91) | 0.94 (0.41-2.13) | 0.30* (0.08-1.20) |
| Women's Health (OB-Gyn, IVF, Breast) | 1.18 (0.47-2.94) | 0.74 (0.30-1.80) | 0.45 (0.09-2.15) |
| Oncology | 2.72* (0.94-7.86) | 1.87 (0.57-6.17) | 1.14 (0.24-5.35) |
| Orthopedics, Sports Medicine, & Rehabilitation | 0.71 (0.15-3.25) | 0.62 (0.18-2.17) | 0.24 (0.02-2.51) |
| Ancillary & Clinical Support (Imaging/Pharmacy/Nutrition/CSSD) | 1.70 (0.29-9.89) | 1.51 (0.30-7.63) | 3.39e-7*** (1.35e-8-8.54e-6) |
| Ambulatory & Preventive Services (OPD/Executive Health) | 2.89 (0.80-10.45) | 1.83 (0.50-6.73) | 3.11 (0.34-28.32) |
| Regenerative Medicine/Cell Therapy | 0.87 (0.26-2.85) | 0.67 (0.23-1.94) | 0.16 (3.37e-3-7.29) |
| Other Specialties, Administrative & Non-clinical, & Unknown/Other | 1.42 (0.43-4.67) | 1.64 (0.53-5.09) | 1.74 (0.40-7.63) |
| **Contract type / tenure** |  |  |  |
| <1 year | Ref | Ref | Ref |
| 1–3 years | 0.83 (0.08-9.09) | 0.41 (0.05-3.36) | 0.13 (6.52e-3-2.62) |
| More than 3 years | 0.55 (0.04-7.95) | 0.34 (0.03-4.28) | 0.06 (9.68e-4-3.46) |
| Indefinite-term (open-ended/permanent) | 1.32 (0.13-13.33) | 0.54 (0.07-4.26) | 0.32 (0.02-4.95) |
| **Patient care involvement / patient severity** |  |  |  |
| Provides care for Level I severity | Ref | Ref | Ref |
| Provides care/treatment for patient severity: Option 2 | 0.84 (0.46-1.55) | 1.42 (0.74-2.71) | 2.42 (0.84-6.99) |
| Does not participate in patient care | 0.98 (0.45-2.16) | 0.80 (0.36-1.80) | 1.00 (0.25-4.04) |
| **Average weekly working hours** |  |  |  |
| <48 hours/week | Ref | Ref | Ref |
| 48 hours/week | 1.01 (0.36-2.84) | 1.00 (0.39-2.53) | 1.83 (0.32-10.57) |
| > 48 hours/week | 1.15 (0.40-3.28) | 0.85 (0.31-2.29) | 1.49 (0.25-8.76) |
| **Working days per week** |  |  |  |
| <5.5 days/week | Ref | Ref | Ref |
| 5.5 days/week | 1.14 (0.44-2.94) | 0.92 (0.37-2.27) | 0.95 (0.22-4.20) |
| > 5.5 days/week | 0.66 (0.29-1.51) | 0.70 (0.31-1.58) | 0.53 (0.16-1.74) |
| **Overtime hours per week** |  |  |  |
| 0 h/wk | Ref | Ref | Ref |
| >0–<12 h/wk | 1.77 (0.62-5.07) | 1.37 (0.49-3.85) | 1.32 (0.31-5.64) |
| 12–<24 h/wk | 2.05* (0.89-4.69) | 1.28 (0.59-2.82) | 1.32 (0.35-4.99) |
| 24–<36 h/wk | 1.93 (0.76-4.89) | 1.38 (0.60-3.20) | 2.35 (0.77-7.17) |
| 36–<48 h/wk | 2.62 (0.66-10.36) | 0.95 (0.28-3.23) | 2.27 (0.28-18.7) |
| ≥48 h/wk | 3.73** (1.13-12.3) | 3.25 (0.79-13.33) | 0.63 (0.03-14.97) |
| Unclear/Not applicable | 1.81 (0.56-5.86) | 2.26 (0.62-8.27) | 2.82 (0.31-25.77) |
| **On-call / additional duty hours per week** |  |  |  |
| 0 h/wk | Ref | Ref | Ref |
| >0–<5 h/wk | 0.73 (0.32-1.69) | 1.05 (0.50-2.22) | 0.43 (0.12-1.49) |
| 5–<10 h/wk | 0.53 (0.24-1.21) | 1.22 (0.56-2.67) | 0.86 (0.27-2.77) |
| 10–<12 h/wk | 0.73 (0.22-2.38) | 1.43 (0.52-3.97) | 0.57 (0.06-5.31) |
| 12–<24 h/wk | 0.71 (0.29-1.76) | 0.95 (0.40-2.23) | 0.99 (0.29-3.45) |
| 24–<36 h/wk | 0.30 (0.07-1.29) | 1.36 (0.25-7.38) | 0.71 (0.05-10.42) |
| ≥48 h/wk | 8.92e-8*** (9.21e-9-8.64e-7) | 1.52e-7*** (2.25e-8-1.03e-6) | 3.37e-7*** (1.96e-8-5.78e-6) |
| **Other/CME training hours in past 12 months** |  |  |  |
| 0-<12 h/year | Ref | Ref | Ref |
| 12–<24 h/year | 0.23 (0.01-4.18) | 0.50 (0.07-3.45) | 1.38 (0.02-87.21) |
| 24–<36 h/year | 0.03** (1.82e-3-0.62) | 0.08** (0.01-0.60) | 0.15 (1.28e-3-17.65) |
| ≥36 h/year | 0.09* (7.27e-3-1.13) | 0.14** (0.03-0.68) | 0.02* (3.47e-4-1.20) |
| No/Unclear | 0.09* (7.80e-3-1.07) | 0.11*** (0.03-0.48) | 0.05 (8.08e-4-2.96) |
| **Fit with job role** |  |  |  |
| Not suitable (4-5) | Ref | Ref | Ref |
| Suitable (1–3) | 0.66 (0.31-1.39) | 0.55 (0.24-1.24) | 0.74 (0.22-2.44) |
| **Fit with income** |  |  |  |
| Inadequate (4-5) | Ref | Ref | Ref |
| Adequate (1–3) | 0.70 (0.38-1.31) | 1.10 (0.55-2.22) | 0.43 (0.15-1.24) |
| **Perceived work pressure (past 3 months)** |  |  |  |
| Very high | Ref | Ref | Ref |
| High | 0.53 (0.24-1.19) | 0.34*** (0.17-0.69) | 0.38* (0.13-1.16) |
| Moderate/Low | 0.35** (0.14-0.91) | 0.24*** (0.11-0.53) | 0.14*** (0.04-0.47) |
| **Relationship with colleagues** |  |  |  |
| Average/Not good | Ref | Ref | Ref |
| Good/Very good | 0.40** (0.18-0.92) | 0.89 (0.36-2.19) | 0.44 (0.10-1.88) |
| **Conflicts with colleagues (frequency)** |  |  |  |
| None | Ref | Ref | Ref |
| Almost never | 2.65e-7*** (2.71e-8-2.60e-6) | 0.90 (0.19-4.36) | 5.45e-7*** (3.39e-8-8.75e-6) |
| Rarely | 1.09 (0.50-2.37) | 1.46 (0.71-3.00) | 1.56 (0.55-4.40) |
| Occasionally | 1.78 (0.81-3.87) | 1.47 (0.71-3.04) | 1.57 (0.43-5.78) |
| Often | 1.71 (0.14-21.66) | 1.32 (0.20-8.64) | 0.33 (9.53e-3-11.16) |
| **Relationship with line manager/supervisor** |  |  |  |
| Good/Very good | Ref | Ref | Ref |
| Average/Not good | 0.73 (0.28-1.87) | 0.83 (0.34-1.99) | 0.47 (0.12-1.87) |
| No answer | Omitted | Omitted | Omitted |
| **Conflicts with supervisor (frequency)** |  |  |  |
| None | Ref | Ref | Ref |
| Almost never | 4.46 (0.51-39.41) | 0.68 (0.09-5.30) | 6.20e-7*** (2.77e-8-1.39e-5) |
| Rarely | 2.93** (1.17-7.31) | 1.86 (0.74-4.67) | 3.19* (0.87-11.73) |
| Occasionally | 4.22** (1.25-14.3) | 1.13 (0.32-4.02) | 5.12 (0.41-63.73) |
| **Relationship with patients** |  |  |  |
| Good/Very good | Ref | Ref | Ref |
| Average/Not-good/Very-not-good | 0.31 (0.07-1.39) | 1.34 (0.31-5.90) | 0.81 (0.08-8.49) |
| No answer | Omitted | Omitted | Omitted |
| **Conflicts with patients (frequency)** |  |  |  |
| None | Ref | Ref | Ref |
| Almost never | 4.45** (1.20-16.45) | 1.61 (0.33-7.77) | 2.21 (0.39-12.65) |
| Rarely | 0.96 (0.44-2.09) | 0.93 (0.40-2.17) | 1.05 (0.30-3.70) |
| Occasionally | 0.68 (0.27-1.73) | 0.49 (0.15-1.61) | 0.81 (0.20-3.31) |
| **Job satisfaction** |  |  |  |
| Neutral/Not satisfied | Ref | Ref | Ref |
| Satisfied (4–5) | 0.39*** (0.19-0.79) | 0.57* (0.31-1.03) | 0.35** (0.13-0.89) |
| **Job stability** |  |  |  |
| Not stable | Ref | Ref | Ref |
| Stable (4–5) | 0.52** (0.28-0.94) | 0.60* (0.35-1.03) | 1.63 (0.63-4.25) |
| Observations: 478 |  |  |  |
| *** p<0.01, ** p<0.05, * p<0.1 |  |  |  |

***Notes:*** *Reference categories are indicated in the table as “Ref”; Categories labelled “(omitted)” were omitted due to collinearity by the estimation routine; Some parameters may be unstable in the presence of quasi-complete separation (see Stata notes: “observations completely determined”). Interpret such estimates with caution.*
